# Murine sepsis phenotypes and differential treatment effects in a randomized trial of prompt antibiotics and fluids

**DOI:** 10.1186/s13054-019-2655-7

**Published:** 2019-11-28

**Authors:** Christopher W. Seymour, Samantha J. Kerti, Anthony J. Lewis, Jason Kennedy, Emily Brant, John E. Griepentrog, Xianghong Zhang, Derek C. Angus, Chung-Chou H. Chang, Matthew R. Rosengart

**Affiliations:** 1 Departments of Critical Care Medicine Emergency Medicine, University of Pittsburgh School of Medicine, 3550 Terrace St, Scaife Hall, #639, Pittsburgh, PA 15261 USA; 2 Clinical Research, Investigation, and Systems Modeling of Acute Illness Center (CRISMA), University of Pittsburgh School of Medicine, Pittsburgh, USA; 3 Department of Emergency Medicine, University of Pittsburgh School of Medicine, Pittsburgh, USA; 4 Department of Surgery, University of Pittsburgh School of Medicine, Pittsburgh, USA; 5 Department of Biostatistics, University of Pittsburgh School of Medicine, Pittsburgh, USA

**Keywords:** Sepsis, Animal model, Antibiotics, Phenotype

## Abstract

**Background:**

Clinical and biologic phenotypes of sepsis are proposed in human studies, yet it is unknown whether prognostic or drug response phenotypes are present in animal models of sepsis. Using a biotelemetry-enhanced, murine cecal ligation and puncture (CLP) model, we determined phenotypes of polymicrobial sepsis prior to physiologic deterioration, and the association between phenotypes and outcome in a randomized trial of prompt or delayed antibiotics and fluids.

**Methods:**

We performed a secondary analysis of male C57BL/6J mice in two observational cohorts and two randomized, laboratory animal experimental trials. In cohort 1, mice (*n* = 118) underwent biotelemetry-enhanced CLP, and we applied latent class mixed models to determine optimal number of phenotypes using clinical data collected between injury and physiologic deterioration. In cohort 2 (*N* = 73 mice), inflammatory cytokines measured at 24 h after deterioration were explored by phenotype. In a subset of 46 mice enrolled in two trials from cohort 1, we tested the association of phenotypes with the response to immediate (0 h) vs. delayed (2 to 4 h) antibiotics or fluids initiated after physiologic deterioration.

**Results:**

Latent class mixture modeling derived a two-class model in cohort 1. Class 2 (*N* = 97) demonstrated a shorter time to deterioration (mean SD 7.3 (0.9) vs. 9.7 (3.2) h, *p* < 0.001) and lower heart rate at 7 h after injury (mean (SD) 564 (55) vs. 626 (35) beats per minute, *p* < 0.001). Overall mortality was similar between phenotypes (*p* = 0.75). In cohort 2 used for biomarker measurement, class 2 mice had greater plasma concentrations of IL6 and IL10 at 24 h after CLP (*p* = 0.05). In pilot randomized trials, the effects of sepsis treatment (immediate vs. delayed antibiotics) differed by phenotype (*p* = 0.03), with immediate treatment associated with greater survival in class 2 mice only. Similar differential treatment effect by class was observed in the trial of immediate vs. delayed fluids (*p* = 0.02).

**Conclusions:**

We identified two sepsis phenotypes in a murine cecal ligation and puncture model, one of which is characterized by faster deterioration and more severe inflammation. Response to treatment in a randomized trial of immediate versus delayed antibiotics and fluids differed on the basis of phenotype.

## Background

Not all septic patients present the same [1], and there is profound variability in the signs and symptoms of overwhelming infection. A “one size fits all” approach to treatment, as recommended by international clinical practice guidelines and state and national healthcare policy, ignores this heterogeneity across patients [2]. Even recent clinical trials in sepsis are agnostic to the variable host immune response, offending pathogens, and patterns of organ dysfunction hidden inside trial populations [3, 4]. As studies of novel sepsis therapeutics continue to be neutral, future gains may come from clinical trials that enrich for groups of sepsis patients that share clinical or biologic characteristics, termed phenotypes, and explore for differences in treatment effects by phenotype [5, 6].

To support this agenda in precision treatment, recent adult and pediatric human cohort studies propose biologic and clinical phenotypes of sepsis that are both prognostic of outcome and predictive of treatment response [7–11]. Yet, there is no animal model of sepsis phenotypes on which to test candidate therapeutics or explore for underlying mechanisms. Prior examples in murine models have used a supervised, biomarker-stratified approach, where treatment effects are explored across groups stratified by baseline values of a candidate biomarker (e.g., IL-6) [12]. Others have explored therapies across groups stratified by heart rate, a predictor of mortality in murine sepsis models [13]. Finally, sepsis severity scoring systems have been developed, but these are subjective and the validity as platforms for the testing of new agents remains to be determined [14].

Latent class mixture modeling is an approach to find the best fitting, optimal number of subclasses in longitudinal data, independent of the relationship with outcome. Latent class-based methods are applied in patients with acute respiratory distress syndrome (ARDS) to identify subphenotypes that are predictive of response to fluid management strategies [15]. We sought to capitalize on a recent murine randomized trial of prompt vs. delayed antibiotics and fluids enhanced by biotelemetry monitoring [16, 17] and use latent class methods to (i) identify murine sepsis phenotypes prior to overt, physiologic deterioration, (ii) explore their biologic profiles, and (iii) test their association with response to treatment. We hypothesized these data would provide “proof-of-concept” that drug-response phenotypes exist in a commonly used preclinical model of sepsis.

## Methods

All experiments were approved by the University of Pittsburgh Institutional Animal Care and Use Committee (PRO13021581) in accordance with guidelines established by the National Institutes of Health.

### Study design

The primary objective of this study was to determine the feasibility of identifying distinct murine sepsis phenotypes using clinical data from biotelemetry-enhanced cecal ligation and puncture (CLP) prior to physiologic deterioration. This period was chosen to mimic the clinical patient course, physiology, and treatment responses found during the early phases of sepsis. We described clinical outcomes of phenotypes, biomarkers of the host response, and tested the association of phenotypes with differential treatment effects in randomized trial of immediate versus delayed antibiotics and fluids.

### Experimental animals

Male C57BL/6 mice (Jackson Laboratories, Bar Harbor, ME) aged 8 to 12 weeks (mass 25–30 g) were used for all experiments. Mice were housed in specific pathogen-free rooms under 12-h light/12-h dark conditions with an ambient temperature of 23 °C ± 1 °C. Mice were allowed to acclimate to their new surroundings for 1 week prior to any experimentation. Animals were given ad libitum access to water and LabDiet Prolab Isopro RMH 3000 diet pellets (LabDiet, St. Louis, MO). To account for circadian variation, we initiated experiments in the morning between 0700 and 1000.

### Cecal ligation and puncture with biotelemetry monitor implantation

A 1-cm ligation, 21-gauge double puncture model of cecal ligation and puncture (CLP) under anesthesia was performed as previously described [16]. At the time of CLP, an HD-X11 wireless biotelemetry device (Data Sciences International, St. Paul, MN) capable of continuously measuring heart rate, core temperature, and activity was implanted into the peritoneal cavity in accordance with manufacturer’s instructions. At the conclusion of the procedure, all mice received a subcutaneous injection of warmed 0.9% normal saline (30 mL/kg) to compensate for intraoperative fluid losses and recreate the hyperdynamic state associated with early sepsis [18]. Analgesia was achieved with buprenorphine (0.1 mg/kg SQ Q6H-Q12H) for 7 days after CLP, as needed. After surgery, each animal was placed in an individual cage on a heating pad until full emergence from anesthesia (animal upright, mobile, foraging activity). The cage was then removed from the heating pad, and biotelemetry monitoring commenced, capturing data each minute. Data collection and analysis was performed using Ponemah 5.20 (Data Sciences International, St. Paul, MN). For animals in non-survival biomarker experiments, euthanasia was carried out at the specified time point with blood obtained from cardiac puncture.

### Clinical data for murine phenotypes in cohorts 1 and 2

We previously developed and validated criteria that define acute physiologic deterioration after cecal ligation and puncture (CLP): (1) 10% decline of heart rate from its peak value and (2) a scaled 10% drop in core temperature calculated as 10% × (peak core temperature − 25 °C) [16]. After CLP, each animal was continuously monitored for changes in heart rate and temperature, and the moment of acute physiologic deterioration was documented. Data for phenotype derivation included only physiologic measurement prior to deterioration. Three clinical variables were included in derivation models for phenotypes: (1) hourly measurements of heart rate (beats per minute), (ii) hourly measurement of temperature (degrees Celsius), and (3) the total time (min) from CLP to acute physiologic deterioration.

### Inflammatory cytokine assays in cohort 2

In a separate cohort of mice prepared under same conditions for biomarker sampling (*N* = 73), heparinized blood was drawn at 24 h after CLP. Blood was centrifuged at 2000*g* for 15 min, and the plasma was frozen at − 80 °C for future analysis. Plasma concentrations of IL-6, IL-10, and TNF-α were analyzed by ELISA (R&D Systems, Minneapolis, MN).

### Randomized trials of antibiotics and fluids

After physiologic deterioration threshold was met, a subset of 46 mice in cohort 1 were enrolled in two parallel randomized trials. These data are previously reported [16]. The first trial randomized 26 mice to receive antibiotics at two different time points: (i) 13 mice immediately at deterioration vs. (ii) 13 mice either 2 or 4 h later (combined as a single arm in this analysis). The second trial randomized 20 mice to receive fluids alone at two time points: (i) 10 mice immediately at deterioration vs. (ii) 10 mice either 2 or 4 h later (combined as a single arm in this analysis). In the previous report of the trial, we observed no significant survival benefit among mice receiving antibiotics immediately at either 2 or 4 h. For this reason and the small sample size, mice treated at the 2 and 4 h timepoints were combined into a single “delayed” group. The experiment continued until the death of the animal or 7 days.

Antibiotic treatment was a single intraperitoneal (IP) dose (25 mg/kg) of imipenem/cilastatin administered in a small volume of sterile 0.9% saline (0.15 mL) to minimize lavage of the peritoneal cavity or any potential contribution to additional fluid resuscitation. Fluid resuscitation consisted of 30 mL/kg 0.9% normal saline, injected subcutaneously.

### Statistical analysis

We determined murine phenotypes using clinical data prior to physiologic deterioration with latent class mixture models. We used three class-defining variables: (1) hourly measurements of heart rate (beats per minute), (2) hourly measurement of temperature (degrees Celsius), and (3) the total time (min) from CLP to acute physiologic deterioration. Latent class mixture models address issues that arise when longitudinal data is too complex for a linear mixed model framework. These models extend linear mixed models in two key ways: (i) several longitudinal outcomes may be collected and (ii) non-observed heterogeneity may exist in the population. We used a linear transformation to link the latent process to the scale of the observed longitudinal variables. We optimized the number of classes using three criteria: (i) Bayesian Information Criterion (BIC), (ii) clinical significance, and (iii) class size. We prioritized the lowest BIC, followed by clinical significance and adequate class size.

We compared clinical and biomarker characteristics between phenotypes using ANOVA, specifically to test for a difference in mean heart rate and temperature at 5 and 7 h after CLP and inflammatory cytokines at 24 h. We compared the time to death and time to deterioration from CLP (in min). The Bonferroni correction was used to adjust for multiple comparisons.

To determine if there was a differential treatment effect between phenotypes, we used Kaplan Meier curves, created separately for mice randomized to treatment at immediate vs. delayed time point. We compared survival using the Wilcoxon test with the Bonferroni correction to adjust for multiple comparisons. We tested for class by treatment time point interaction using Cox proportional hazards models. The proportional hazards assumption was checked with a log-log survival plot. Gray’s model was used as a comparison analysis when the proportional hazards assumption was violated.

All tests were two-sided with *α* = 0.05. Statistical analysis was conducted using Stata 14 (StataCorp, College Station, TX, USA), R version 3.2.3 package LCMM and SAS 9.3 (SAS Institute, Cary, NC, USA).

## Results

### Clinical features of cohorts

The median time from CLP to physiologic deterioration was a 452 min [IQR 412, 496 min] in cohort 1 and a median 446 min [IQR 411, 489 min] in cohort 2 for biomarker measurement. The median time to death was 3124 min [IQR 1724, 4059 min] in cohort 1.

### Latent class mixture modeling: determination of the number of phenotypes

In cohort 1, latent class mixture models suggested a two-class (*k* = 2) model best balanced optimal fit, clinical significance, and class size. Specifically, the Bayesian information criterion was lowest in *k* = 3 (11,356) and *k* = 4 (11,348) compared to *k* = 2 (11372), suggesting more model complexity provided better optimal fit. However, the *k* = 3 and *k* = 4 models produced classes with two mice, respectively. Although the decrease in BIC would favor the inclusion of additional classes, we retained a two-class model due to the small number of subjects and lack of clinical significance in more complex models. We refer to final model members as class 1 (*N* = 21, 17%) and class 2 (*N* = 97, 82%). The latent class probability of class membership in class 1 was > 0.8 in 86% of mice compared to 91% of members in class 2. These probabilities suggest moderate to strong likelihood of class assignment.

### Clinical and biologic characteristics of each phenotype in cohort 1

**Fig. 1:**
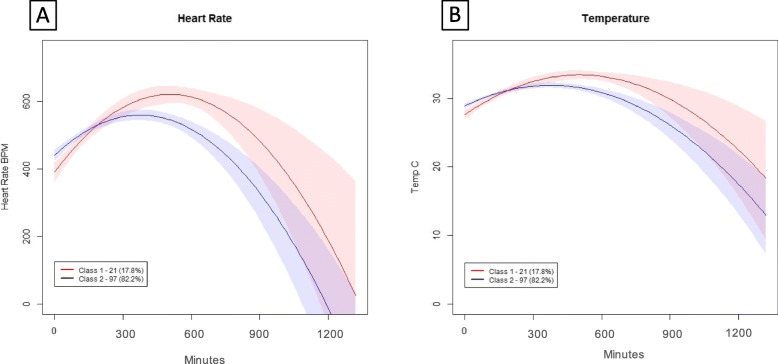
Comparison of trajectory clinical characteristics after CLP for class 1 (*N* = 21, *red*) vs. class 2 (*N* = 97, *blue)*. **a** Heart rate (bpm) and **b** temperature (°C)

**Fig. 2:**
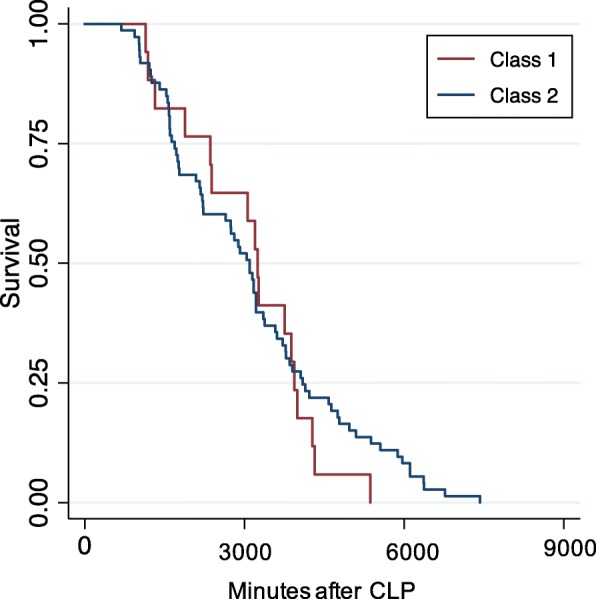
Kaplan Meier curve showing similar survival comparing class 1 (*N* = 21, *red*) to class 2 (*N* = 97, *blue*) mice after CLP

As shown in Table 1, class 2 was defined by a shorter mean time to meeting physiologic deterioration compared to class 1 (7.3 (SD 0.9) h vs. 9.7 (SD 3.2) h, *p* < 0.01). To illustrate the behavior of latent class clinical characteristics, Fig. 1 demonstrates the trajectory of mean heart rate and temperature prior to deterioration. For both variables, on average, class 2 mice had a lower heart rate and temperature compared to class 1. For example, at 7 h after CLP, the mean heart rate was 564 beats per min (SD 55) in class 2 compared to 626 (SD 35) beats per minute for class 1 (*p* < 0.01). We observed no difference in overall time to survival among mice in class 2 vs. class 1 (Table 1, Fig. 2, log rank *p* = 0.75).

**Table 1:** Comparison of key clinical characteristics across phenotypes (*N* = 118)

Characteristic	All mice	Class 1	Class 2	*p* value^b^
No.	118	21	97	
Time to deterioration [h]: mean (SD)	7.7 (1.8)	9.7 (3.2)	7.3 (0.9)	< 0.001
Heart rate 5 h after CLP [bpm]: mean (SD)	590 (79)	592 (108)	589 (71)	0.87
Temperature 5 h after CLP [°C]: mean (SD)	32.3 (1.7)	32.7 (1.4)	32.2 (1.7)	0.17
Heart rate 7 h after CLP [bpm]: mean (SD)	580 (58)	626 (35)	564 (55)	< 0.001
Temperature 7 h after CLP [°C]: mean (SD)	32.1 (1.7)	33.5 (1.3)	31.6 (1.6)	< 0.001
Time to death^a^ [h]: mean (SD)	52.5 (26.3)	51.6 (20.3)	52.7 (27.6)	0.88

*Abbreviations*: *No.* number, *h* hours, *SD* standard deviation, *CLP* cecal ligation and puncture, *bpm* beats per minute

^a^Time to death available in 17 mice in class 1 and 73 mice in class 2

^b^*p* value compares class 1 to class 2 using ANOVA

**Fig. 3:**
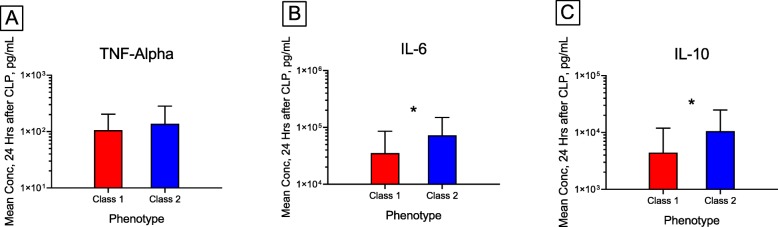
Comparison of inflammatory cytokines measured at 24 h after CLP for mice in class 1 (*N* = 64, *red*) and class 2 (*N* = 9, *blue*). **a** TNF, **b** IL-6, and **c** IL-10. *corresponds to *p* = 0.055 for IL-6 and *p* = 0.046 for IL-10

In cohort 2 for biomarker measurement (*N* = 73), we derived similar phenotypes using latent class mixture models (Table 2). We found 64 mice in class 1 (88%) and 9 mice in class 2 (12%). As shown in Fig. 3, the biomarkers of host response measured at 24 h were greater among class 2 mice. For example, both IL6 (mean (SD): 72,646 (76,547) vs. 35,359 (50,103), *p* = 0.055) and IL10 (mean (SD) 10,588 (14,299) vs. 4418 (7499), *p* = 0.046) were greater in class 2 than in class 1.

**Table 2:** Comparison of key clinical characteristics across phenotypes in subset of mice for biomarker measurement (*N* = 73)

Characteristic	All mice	Class 1	Class 2	*p* value^b^
No.	73	64	9	
Time to deterioration [h]: mean (SD)	7.5 (1.2)	7.7 (1.0)	5.6 (0.9)	< 0.001
Heart rate 5 h after CLP [bpm]: mean (SD)	574 (59)	586 (46)	461 (46)	< 0.001
Temperature 5 h after CLP [°C]: mean (SD)	31.7 (1.5)	32.0 (1.3)	29.3 (1.2)	< 0.001
Heart rate 7 h after CLP^a^ [bpm]: mean (SD)	566 (45)	568 (42)	438 (−)	0.003
Temperature 7 h after CLP^a^ [°C]: mean (SD)	31.7 (1.1)	31.7 (1.0)	29 (−)	< 0.001

*Abbreviations*: *No*. number, *SD* standard deviation, *CLP* cecal ligation and puncture, *bpm* beats per minute

^a^Only 1 mouse with clinical data available at 7 h after CLP

^b^*p* value compares class 1 to class 2 using analysis of variance (ANOVA)

### Treatment effects in two randomized trials

**Fig. 4:**
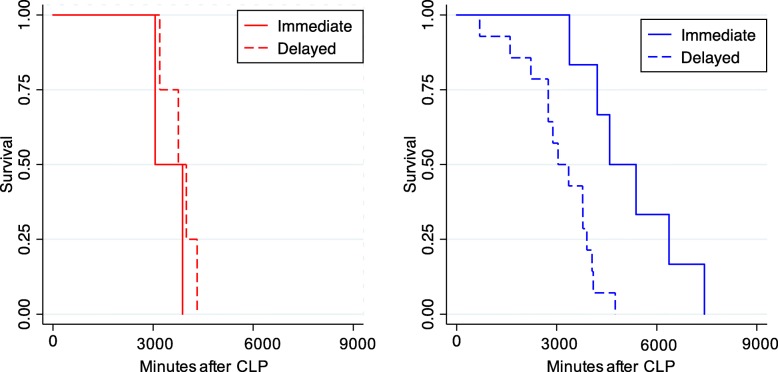
**a** Survival for class 1 mice (*N* = 6, *red*) and **b** class 2 mice (*N* = 20, *blue*) randomized to immediate (*solid*) vs. delayed (*dashed*) antibiotic treatment after CLP. *p* value for interaction = 0.03

**Fig. 5:**
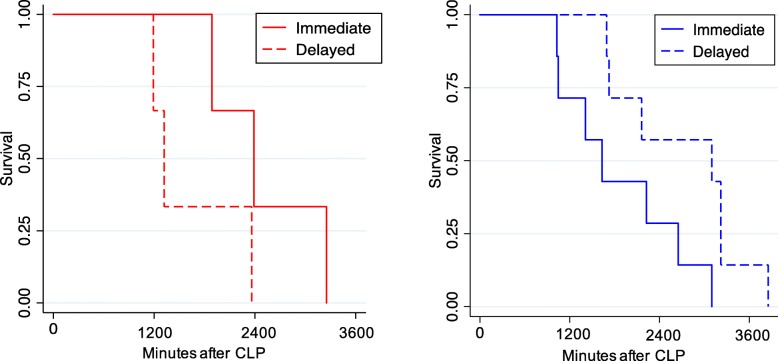
**a** Survival for class 1 mice (*N* = 6, *red*) and **b** class 2 mice (*N* = 14, *blue*) randomized to immediate (*solid*) vs. delayed (*dashed*) fluids after CLP. *p* value for interaction = 0.02

In trial 1, the 8 mice randomized to receive immediate antibiotics compared to 16 mice who received delayed antibiotics (≥ 2 h) had significantly different survival (Wilcoxon *p* = 0.04 for overall comparison of arms, Cox *p* value for interaction = 0.03, Gray’s *p* value = 0.76, Fig. 4). In class 2, mice randomized to receive early antibiotics had a longer survival compared to mice randomized to receive delayed antibiotics (Wilcoxon *p* = 0.02 across arms). In class 1, mice randomized to receive early antibiotics had no difference in survival compared to those who received delayed antibiotics (Wilcoxon *p* = 1.0 across arms). A similar analysis among 20 mice randomized to receive immediate vs. delayed fluids reached statistical significance for treatment by class interaction (Fig. 5, Cox *p* value for interaction = 0.02).

### Sensitivity analyses

First, we derived optimal class number after excluding the time to deterioration variable. When using only heart rate and temperature as class-defining characteristics, we confirmed statistically that *k* = 2 was an optimal class number. The BIC was lowest for *k* = 2 (BIC = 11,394) compared to *k* = 3 (BIC = 11,398) or *k* = 4 (BIC = 11,401). Second, to determine the advantage of understanding predictive phenotypes from unsupervised models prior to deterioration compared to a different measure of trajectory, we stratified mice by above or below median time to deterioration (min) (Additional file 1: Figure S1). We found that comparing treatments across mice above (*N* = 29) to those below the median time to deterioration (*N* = 27) had no evidence of statistical interaction for early antibiotics or fluids (Additional file 1: Figure S2, Cox *p* = 0.9). In this example, the benefit of immediate antibiotics was similar in both groups.

## Discussion

In this biotelemetry-enhanced murine model of polymicrobial sepsis, we demonstrate the feasibility of deriving clinical sepsis phenotypes prior to overt physiologic deterioration. The evidence suggests there are two phenotypes with different trajectory of clinical characteristics and time to deterioration. Most importantly, phenotypes were predictive of response to immediate versus delayed antibiotic and fluid treatment in small randomized trials. These data suggest that clinical sepsis phenotypes may be present in a commonly used animal model of sepsis and serve as a platform to explore heterogeneity of treatment effects for current and novel therapeutics.

There are several possible explanations for the observed heterogenous response. First, previous studies report differential microbial clearance in CLP models of sepsis [18]. Second, photoperiod-dependent or gut microbiome variability in murine sepsis response are reported, but yet to be explored in our models [19]. Finally, variability in technique may result in heterogenous response [20].

Prior work attempted to measure severity among mice with polymicrobial sepsis after CLP, but did not explore data for unsupervised clustering models. The murine sepsis score (MSS) is comprised of seven behavioral and physical characteristics that identify phenotypes representative of progressive stages in the evolution of the septic response [5]. It was developed, in part, to mimic sophisticated scoring systems in human critical illness, such as the Sequential Organ Failure Assessment (SOFA) scores [21]. Using a dose-response cecal slurry model to derive the score, authors report a high discriminatory power for survival, organ injury, and cytokine concentrations and excellent inter-rater reliability [14]. A similar score has been developed for *Klebsiella pneumoniae* pneumonia: the mouse clinical assessment for sepsis (M-CASS) [22]. However, resolution remains modestly subjective and dependent on the frequency of mouse scoring, which can introduce bias from handling. It is unknown how these scores correlate with underlying biology or treatment effects in randomized trials of sepsis treatment.

Inflammatory cytokines may also stratify sepsis in murine models. For example, IL6 measured 6 h post-CLP and dichotomized at a threshold of 2000 pg/mL, effectively classified animals as non-survivors vs. survivors: 90% vs. 30% mortality [23]. Many other prognostic biomarkers are reported after CLP, including TNF-α, KC, MIP-2, IL-1 receptor antagonist, TNF soluble receptor I, and TNF soluble receptor II [12]. And when mice were stratified according to IL6 and survival, experimental therapies such as dexamethasone could be individualized to alter outcome. However, many limitations are present including (i) challenges in blood sampling in small animal models, (ii) timeliness of assay read-outs to inform potential emergency treatments, and (iii) measurement at a single time point after deterioration. These limitations are overcome in the current study by using a clinical phenotyping strategy that incorporates automated biotelemetry, prior to a potential randomization moment early in deterioration, and with short computational time.

A related question is whether or not clinical murine phenotypes have face validity? The class 1 mice were more “fit” insofar that they appeared more tolerant to, or rather, better able to compensate for, the sepsis of CLP. Specifically, these mice exhibited more moderate vital sign changes, delayed clinical deterioration, and less inflammation. In contrast, the predictive class 2 deteriorated quickly, had greater inflammation, and significantly greater perturbation in vital sign parameters. The human correlate to these features are readily identified in multiple human cohort studies, where a subset of septic patients rapidly progress to shock and display unremitting inflammation found on serial cytokine measurements [6, 24]. On the other hand, there are different subsets of patients with seemingly greater tolerance to sepsis, either due to the host immunity to the offending pathogen, the primary organ affected, or underlying characteristics like age and comorbidity. Although reassuring, these comparisons are inherently challenged as murine studies control the onset of sepsis episode, whereas the start of the sepsis episode in humans is variable and typically unknown.

Recent studies in human sepsis also identify a hyperinflammatory subset, similar to our murine class 2 [6]. In ARDS, for which sepsis is the primary risk factor, more than 1 in 3 patients are proposed as members of a hyperinflammatory phenotype using latent class modeling [15, 25]. This group had lower systolic blood pressure, greater acidosis, and higher inflammatory cytokines measured at the time of enrollment in the NHLBI ARDS Network’s randomized controlled trials. In complementary work, intensive care unit patients with sepsis were grouped into phenotypes using genome-wide blood gene expression profiles and consensus clustering. These data proposed four endotypes of sepsis with differential survival, including two endotypes (MARS2 and MARS4) with expression of genes involving pro-inflammatory and innate immune function [9]. Taken together, there is significant heterogeneity in human sepsis which may correlate with the preliminary patterns seen in these murine experiments.

When sepsis phenotypes were determined prior to enrollment in murine randomized trials, we found heterogeneity of treatment effect for broad-spectrum antibiotic therapy and fluids administered at the time of clinical deterioration. Only mice in class 2, which were the less fit, hyperinflammatory group, was there a significantly different survival with immediate vs. delayed therapy. Prior work in large human cohorts has suggested a similar interaction between illness acuity and prompt antibiotic therapy. For example, Liu et al. report that for there was no significant association between delay of antibiotic therapy for more than 3 h compared to before 3 h among a subset of patients with sepsis but no organ dysfunction [26]. Yet, the association was not only present, but strongest, among the sickest patients with septic shock. Similar findings were present in a statewide cohort study of more than 50,000 sepsis patients, yet the statistical test of interaction was not significant [27]. In the future, both human and animal studies should be motivated to explore for treatment by subgroup interactions, where groups are both those with clinically apparent (e.g., shock, gender, community vs. nosocomial infection) and “hidden” phenotypes.

The heterogeneity in response to fluid therapy by class is also not surprising given the conflicting findings in human studies of fluid resuscitation in sepsis. A randomized trial in sub-Saharan Africa found greater mortality among septic patients who were aggressively treated with protocol-guided intravenous fluid vs. usual care [28], similar to the finding in the Fluid Expansion as Supportive Therapy (FEAST) trial among children with impaired perfusion [29]. Large cohort studies demonstrate no association between the timing of completing the initial fluid bolus in sepsis and in-patient mortality [7]. The class by fluid treatment effect in these murine data may suggest that like antibiotic therapy, more attention is needed to precisely tailor fluid administration to specific phenotypes of sepsis.

There are several imitations to this analysis. First, the data derive from a small sample of male mice, which may be underpowered in both the tests of interaction in the randomized trials, proportional hazards assumptions in Cox models, and biomarker analyses. Second, latent class mixture models converged using hourly measurements of heart rate and temperature, but more complex repeated measures of entropy or variation could refine phenotype derivation. Notably, class number was similar in sensitivity analyses with reduced model variables. Third, we recognize that the time to antibiotic treatment effects may be subject to the choice of treatment. We utilized single dose imipenem due to its broad antimicrobial activity and standardized use in CLP models, although data suggest alternatives [30]. This drug may or may not be feasible in human trials, nor its effects generalizable from mice to humans. Fourth, we are also currently limited by the sex, age, and strain of mice used. Different variations on these parameters may lead to different phenotype derivations and subsequent treatment effects. Fifth, the mortality in our model was high; thus, findings may not be generalizable to a lower acuity septic cohort. Sixth, we analyzed survival data linked to time of CLP; however, alternative anchor times could have been chosen. Finally, the results presented are a proof of concept for risk-based or cluster-based classes in murine sepsis. It is plausible the classes represent two distinct groups with variable biology; however, only two parameters were used in the models. Future studies that use multi-parameter distributions to explore whether groups are partitions along a single distribution or distinct clusters are a future direction for murine studies [31].

## Conclusions

In conclusion, we identified two phenotypes before clinical deterioration in murine sepsis due to CLP, one of which is characterized by faster deterioration and more severe inflammation. Response to treatment in a randomized trial of immediate versus delayed antibiotics and fluids differed on the basis of phenotype.

## Supplementary information

**Additional file 1:** Murine sepsis phenotypes and differential treatment effects in a randomized trial of prompt antibiotics and fluids. Supplementary figures (Figure S1 and Figure S2) with accompanying legends.

## Data Availability

All datasets used and/or analyzed during the current study are available from the corresponding author on reasonable request.

## Abbreviations

ARDS: Acute respiratory distress syndrome
BIC: Bayesian Information Criterion
CLP: Cecal ligation and puncture
FEAST: Fluid Expansion as Supportive Therapy
IL: Interleukin
M-CASS: Mouse clinical assessment for sepsis
MSS: Murine sepsis score
SD: Standard deviation
SOFA: Sequential Organ Failure Assessment
TNF: Tumor necrosis factor

## References

[1] Seymour CW, Liu VX, Iwashyna TJ (2016). Assessment of Clinical Criteria for Sepsis: for the Third International Consensus Definitions for Sepsis and Septic Shock (Sepsis-3). JAMA..

[2] Klompas Michael, Calandra Thierry, Singer Mervyn (2018). Antibiotics for Sepsis—Finding the Equilibrium. JAMA.

[3] Opal SM, Laterre PF, Francois B (2013). Effect of eritoran, an antagonist of MD2-TLR4, on mortality in patients with severe sepsis: the ACCESS randomized trial. JAMA.

[4] Investigators P. Early, goal-directed therapy for septic shock - a patient-level meta-analysis. N Engl J Med. 2017. 10.1056/NEJMoa1701380 28320242

[5] Iwashyna TJ, Burke JF, Sussman JB, Prescott HC, Hayward RA, Angus DC (2015). Implications of heterogeneity of treatment effect for reporting and analysis of randomized trials in critical care. AmJ Respir Crit Care Med.

[6] Seymour CW, Kennedy JN, Wang S (2019). Derivation, validation and potential treatment implications of novel clinical phenotypes for sepsis. JAMA.

[7] Sweeney TE, Azad TD, Donato M (2018). Unsupervised analysis of transcriptomics in bacterial sepsis across multiple datasets reveals three robust clusters. Crit Care Med.

[8] Wong Hector R., Sweeney Timothy E., Hart Kimberly W., Khatri Purvesh, Lindsell Christopher J. (2017). Pediatric Sepsis Endotypes Among Adults With Sepsis. Critical Care Medicine.

[9] Scicluna BP, van Vught LA, Zwinderman AH (2017). Classification of patients with sepsis according to blood genomic endotype: a prospective cohort study. Lancet Respir Med.

[10] McHugh L, Seldon TA, Brandon RA (2015). A molecular host response assay to discriminate between sepsis and infection-negative systemic inflammation in critically ill patients: discovery and validation in independent cohorts. PLoS Med.

[11] Wong HR, Cvijanovich NZ, Allen GL (2011). Validation of a gene expression-based subclassification strategy for pediatric septic shock. Crit Care Med.

[12] Osuchowski MF, Connett J, Welch K, Granger J, Remick DG (2009). Stratification is the key: inflammatory biomarkers accurately direct immunomodulatory therapy in experimental sepsis. Crit Care Med.

[13] Kim J, Arnaout L, Remick DG. Hydrocortisone, ascorbic acid and thiamine (HAT) theray decreases oxidative stress, improves cardiovascular function and improves survival in murine sepsis. Shock. 2019. 10.1097/SHK0000000000001385. 10.1097/SHK.0000000000001385 PMC11615833 31169765

[14] Shrum B, Anantha RV, Xu SX (2014). A robust scoring system to evaluate sepsis severity in an animal model. BMC Res Notes.

[15] Famous KR, Delucchi K, Ware LB (2017). Acute respiratory distress syndrome subphenotypes respond differently to randomized fluid management strategy. Am J Respir Crit Care Med.

[16] Lewis AJ, Griepentrog JE, Zhang X, Angus DC, Seymour CW, Rosengart MR (2018). Prompt administration of antibiotics and fluids in the treatment of sepsis: a murine trial. Crit Care Med.

[17] Lewis AJ, Yuan D, Zhang X, Angus DC, Rosengart MR, Seymour CW (2016). Use of biotelemetry to define physiology-based deterioration thresholds in a murine cecal ligation and puncture model of Sepsis. Crit Care Med.

[18] Moitra R, Beal DR, Belikoff BG, Remick DG (2011). Presence of pre-existing antibodies mediate survival in sepsis. Shock..

[19] Kiank C (2007). Seasonal variations in inflammatory responses to sepsis and stress in mice. Crit Care Med.

[20] Hubbard WJ, Choudhry M, Schwacha MG (2005). Cecal ligation and puncture. Shock..

[21] Vincent JL, Moreno R, Takala J (1996). The SOFA (Sepsis-related Organ Failure Assessment) score to describe organ dysfunction/failure. On behalf of the Working Group on Sepsis-Related Problems of the European Society of Intensive Care Medicine. Intensive Care Med.

[22] Huet O, Ramsey D, Miljavec S (2013). Ensuring animal welfare while meeting scientific aims using a murine pneumonia model of septic shock. Shock..

[23] Remick DG, Bolgos GR, Siddiqui J, Shin J, Nemzek JA (2002). Six at six: interleukin-6 measured 6 h after the initiation of sepsis predicts mortality over 3 days. Shock..

[24] Shankar-Hari M, Phillips GS, Levy ML (2016). Developing a new definition and assessing new clinical criteria for septic shock: for the Third International Consensus Definitions for Sepsis and Septic Shock (Sepsis-3). JAMA..

[25] Bos LD, Schouten LR, van Vught LA (2017). Identification and validation of distinct biological phenotypes in patients with acute respiratory distress syndrome by cluster analysis. Thorax..

[26] Liu VX, Fielding-Singh V, Greene JD (2017). The timing of early antibiotics and hospital mortality in Sepsis. Am J Respir Crit Care Med.

[27] Seymour CW, Gesten F, Prescott HC (2017). Time to treatment and mortality during mandated emergency care for sepsis. N Engl J Med.

[28] Andrews B, Muchemwa L, Kelly P, Lakhi S, Heimburger DC, Bernard GR (2014). Simplified severe sepsis protocol: a randomized controlled trial of modified early goal-directed therapy in Zambia. Crit Care Med.

[29] Maitland K, Kiguli S, Opoka RO (2011). Mortality after fluid bolus in African children with severe infection. N Engl J Med.

[30] de Tymowski C, Correia MDT, Monteiro RC, Montravers P, Ben Mkaddem S. Bacterial proliferation may be the key component of sepsis mortality. Infect Immun. 2018; 86Le00948–17. doi:10.1128/IAI.00948-17. 10.1128/IAI.00948-17 PMC6204699 30361458

[31] Ankerst M (1999). OPTICS: Ordering points to identify the clustering structure. SIGMOD.

