# Supplementary material for: Murine sepsis phenotypes and differential treatment effects in a randomized trial of prompt antibiotics and fluids
Source: Crit Care. 2019 Nov 28;23:384. doi: 10.1186/s13054-019-2655-7 (PMC6883631; doi:10.1186/s13054-019-2655-7)
Supplement: Supplementary file 1 — Additional file 1: Murine sepsis phenotypes and differential treatment effects in a randomized trial of prompt antibiotics and fluids. Supplementary figures (Figure S1 and Figure S2) with accompanying legends. [file 13054_2019_2655_MOESM1_ESM.docx]

**Additional file 1**

**Murine sepsis phenotypes and differential treatment effects in a randomized trial of prompt antibiotics and fluids**

Christopher W. Seymour, MD MSc^1-3^; Samantha J. Kerti, MS^1,2^; Anthony J. Lewis, MD MSc^4^; Jason Kennedy, MS^1,2^; Emily Brant, MD,^1,2^ John E. Griepentrog, MD^4^; Xianghong Zhang, PhD^4^; Derek C. Angus, MD MPH^1,2^; Chung-Chou H. Chang, Ph.D^1,2,5^; and Matthew R. Rosengart, MD MPH^1,2,4^

**Figure S1**

Sensitivity analysis comparing the trajectory of heart rate and temperature after CLP for Class 1 (N=36, *red*) vs. Class 2 (N=82, *blue)* derived using latent class mixture models that do not include the time-to-deterioration variable. Panel A shows heart rate (bpm) and Panel B shows temperature (°C).

**Figure S2**

Sensitivity analysis of heterogeneity of treatment effects. Panel A shows survival for mice with greater than the median time to deterioration (N=29, *red*) and Panel B shows survival for mice less than the median time to deterioration (N=27, *blue*) who were subsequently randomized to immediate (*solid*) vs. delayed (*dashed*) antibiotics after CLP. P value for interaction = 0.9, as both groups had improved survival when randomized to immediate treatment.
